# Familial Hypocalciuric Hypercalcemia Type 1 and Autosomal-Dominant Hypocalcemia Type 1: Prevalence in a Large Healthcare Population

**DOI:** 10.1016/j.ajhg.2020.04.006

**Published:** 2020-05-07

**Authors:** Ridge Dershem, Caroline M. Gorvin, Raghu P.R. Metpally, Sarathbabu Krishnamurthy, Diane T. Smelser, Fadil M. Hannan, David J. Carey, Rajesh V. Thakker, Gerda E. Breitwieser, Regeneron Genetics Center

**Affiliations:** 1 Molecular and Functional Genomics, Weis Center for Research, Geisinger, Danville, PA 17822, USA; 2 Academic Endocrine Unit, Radcliffe Department of Medicine, University of Oxford, Oxford OX3 9DU, UK; 3 Nuffield Department of Women’s & Reproductive Health, University of Oxford, Oxford OX3 9DU, UK; 4 Regeneron Genetics Center, Tarrytown, NY 10591, USA

**Keywords:** familial hypocalciuric hypercalcemia type 1, autosomal-dominant hypocalcemia type 1, calcium sensing receptor, genomics, disease prevalence, serum calcium, disease associations, Sequence Kernel Association Test, SKAT

## Abstract

The calcium-sensing receptor (CaSR) regulates serum calcium concentrations. *CASR* loss- or gain-of-function mutations cause familial hypocalciuric hypercalcemia type 1 (FHH1) or autosomal-dominant hypocalcemia type 1 (ADH1), respectively, but the population prevalence of FHH1 or ADH1 is unknown. Rare *CASR* variants were identified in whole-exome sequences from 51,289 de-identified individuals in the DiscovEHR cohort derived from a single US healthcare system. We integrated bioinformatics pathogenicity triage, mean serum Ca concentrations, and mode of inheritance to identify potential FHH1 or ADH1 variants, and we used a Sequence Kernel Association Test (SKAT) to identify rare variant-associated diseases. We identified predicted heterozygous loss-of-function *CASR* variants (6 different nonsense/frameshift variants and 12 different missense variants) in 38 unrelated individuals, 21 of whom were hypercalcemic. Missense *CASR* variants were identified in two unrelated hypocalcemic individuals. Functional studies showed that all hypercalcemia-associated missense variants impaired heterologous expression, plasma membrane targeting, and/or signaling, whereas hypocalcemia-associated missense variants increased expression, plasma membrane targeting, and/or signaling. Thus, 38 individuals with a genetic diagnosis of FHH1 and two individuals with a genetic diagnosis of ADH1 were identified in the 51,289 cohort, giving a prevalence in this population of 74.1 per 100,000 for FHH1 and 3.9 per 100,000 for ADH1. SKAT combining all nonsense, frameshift, and missense loss-of-function variants revealed associations with cardiovascular, neurological, and other diseases. In conclusion, FHH1 is a common cause of hypercalcemia, with prevalence similar to that of primary hyperparathyroidism, and is associated with altered disease risks, whereas ADH1 is a major cause of non-surgical hypoparathyroidism.

## Introduction

The calcium-sensing receptor (CaSR), encoded by *CASR* on chromosome 3q21.1, is a 1,078 amino acid class C G protein-coupled receptor that is highly expressed in calcitropic tissues including parathyroid glands and kidneys.^1^ CaSR has an extracellular amino-terminal domain which binds Ca^2+^ (ECD, residues 1–612), a heptahelical transmembrane domain (TMD, residues 613–862), and a cytoplasmic carboxyl terminal domain (CT, residues 863–1078).^2^ CaSR couples to heterotrimeric G proteins (Gα_11_) and the adaptor related protein complex-2 σ-subunit (AP2σ) to mediate signaling via intracellular Ca^2+^ (Ca^2+^_i_) mobilization and mitogen-activated protein kinases.^3^^,^^4^ The importance of CaSR-mediated signaling for Ca^2+^ homeostasis is confirmed by germline loss-of-function mutations in *CASR* (MIM: 145980; 239200), *GNA11* (MIM: 145981), and *AP2S1* (MIM: 600740) that cause familial hypocalciuric hypercalcemia types 1–3 (FHH1-3), respectively. FHH1 accounts for ∼65% of cases, and is an autosomal-dominant condition characterized by lifelong elevations of serum calcium concentrations and normal or elevated serum parathyroid hormone (PTH) concentrations.^5^ FHH1 is usually asymptomatic and requires no intervention,^5^ although it has a serum biochemical profile similar to primary hyperparathyroidism (PHPT), which is typically treated by parathyroidectomy. Distinguishing FHH1 from PHPT, generally done by assessing urinary Ca^2+^ excretion, i.e., ∼80% of FHH-affected individuals are hypocalciuric (Ca creatinine clearance ratio [CCCR] < 0.01) versus <20% of PHPT individuals with CCCR < 0.01,^6^^,^^7^ is required to prevent FHH1-affected individuals from undergoing unnecessary parathyroid surgery.

In contrast, germline gain-of-function mutations of *CASR* (MIM: 601198) and *GNA11* (MIM: 615361) cause autosomal-dominant hypocalcemia types 1–2 (ADH1-2), respectively.^8^ ADH1 accounts for ∼70% of ADH cases^9^ and has a biochemical phenotype opposite of FHH1, i.e., individuals have low serum Ca concentrations, normal or low PTH concentrations, relative or absolute hypercalciuria, and may have symptomatic hypocalcemia and ectopic calcifications affecting the kidneys and/or basal ganglia.^8^ FHH1 and ADH1 are considered rare disorders, and may be diagnosed following incidental biochemical testing of asymptomatic individuals, or in the case of ADH1, following the presentation of an individual with symptomatic hypocalcemia.^10^, ^11^, ^12^ However, the prevalence of FHH1 or ADH1 remains to be determined in the general population.^10^, ^11^, ^12^, ^13^, ^14^

The major goal of this study was to use whole-exome sequencing and clinical laboratory data from a single large US health system to identify individuals with FHH1 and ADH1 and to estimate the population frequencies of these rare disorders. To achieve this, we combined rare variant pathogenicity triage with serum Ca measures from the electronic health record (EHR) and verified clinically identified potential FHH1 or ADH1 individuals by heterologous expression and functional analyses of predicted pathogenic variants. Clinical validation of FHH1- and ADH1-associated variants was further bolstered by *in silico* pedigree analysis of individuals harboring rare *CASR* variants. The broad expression of CaSR in cells and tissues that do not directly contribute to serum Ca^2+^ homeostasis argues that FHH1-affected individuals may have altered risks of non-calcitropic diseases. However, systematic assessment of these potential risks in FHH1 variant carriers has not been possible to date due to the small numbers of individuals identified in most FHH1 pedigrees. We therefore capitalized on the numbers of FHH1-affected individuals identified in this cohort to apply an unbiased, rare variant binning approach to examine the non-calcitropic disorders associated with elevated serum Ca concentrations and/or reduced CaSR function.

## Subjects and Methods

Supplemental Methods contains additional methodological details.

### DiscovEHR Cohort

The initial Geisinger cohort consisted of 51,289 individuals, including 563 individuals below the age of 18, who consented to participate in the MyCode Community Health Initiative,^15^ and whose germline DNA underwent whole-exome sequencing (WES) by Regeneron Genetics Center (Table S1).^16^ The DiscovEHR cohort was predominantly of northern European descent (50,387 individuals), plus 57 American Indian/Alaska Native; 137 Asian; 570 Black/African American; 40 Native Hawaiian/Pacific Islander; and 3 Uncategorized/Other, as self-reported in the EHR.

### Clinical Data

All recorded clinical and biochemical measurements and unique International Classification of Disease-9 (ICD9) codes for each individual were extracted from the EHR in a de-identified manner through an approved data broker in accordance with Institutional Review Board approvals. Medians and means of all available outpatient serum and urinary biochemical and clinical parameters were analyzed. For individuals with available albumin concentrations, serum calcium (denoted as serum Ca) was adjusted for serum albumin concentration as follows: adjusted Ca (Ca_alb_) = total serum Ca (mg/dL) + 0.8^∗^(4-serum albumin (mg/dL)). At least one serum Ca measure was available in the EHR for 50,208 of 51,289 individuals in the cohort (∼98%). When available in the EHR, we report intact PTH levels, measured as part of routine care by electrochemiluminescence at Geisinger Medical Laboratories, with a reference interval of 15-65 pg/mL. To determine the contribution of carriers of rare *CASR* hypercalcemia-associated variants to the fraction of patients having overt hypercalcemia, i.e., mean serum Ca ≥ 10.2 mg/dL, we identified the subset of individuals in the cohort not treated with either cinacalcet or vitamin D and with at least 5 measures of serum Ca in their EHR as an indicator of persistent hypercalcemia. Likewise, we determined the number of individuals having persistent hypocalcemia that were not treated with either cinacalcet or vitamin D and had at least 5 serum Ca measures and mean serum Ca of ≤ 8.5 mg/dL.

### Exome Sequencing

Exome sequencing, alignment, quality control, and variant calling were performed as previously described.^16^ Sanger DNA sequencing was used to confirm all nonsense, frameshift, and missense rare variants with likely clinical impact.

### Segregation Analysis

To leverage the power of familial transmission as a means to determine clinical significance of rare genetic variants, we compared clinical laboratory data for related and unrelated individuals who were carriers of the same rare FHH1- or ADH1-associated variant. To increase the probability of identifying close relations, we used a larger DiscovEHR cohort of 92,816 individuals.^17^ Relatedness was determined by genome-wide estimation of shared identity by descent (IBD) using PRIMUS (Pi-HAT scores provided in relevant tables). We identified close relations (parent-child; full sibling; 2° relations) and their serum Ca phenotypes whether or not they were carriers of the variant.

### Association Studies

We performed a binned genetic association analysis of *CASR* variants to identify associated clinical traits. A large-scale simulation study of DiscovEHR data demonstrated that Sequence Kernel Association Test (SKAT analysis) for case numbers ≥ 200, assuming unbalanced case-control ratios (as expected for different ICD9 codes), resulted in >90% power to detect associations and had well-controlled type I error.^18^ We combined all individuals with rare variants (nonsense plus frameshift plus functionally confirmed missense loss-of-function variants) and performed SKAT analysis using default parameters against all ICD9 codes with a minimum of 200 individuals in the cohort, regardless of genotype, adjusted for sex, age, age^2^, and first four principal components (SKAT R package).

### Variant Analysis

Variants were identified relative to the longest *CASR* transcript (transcript 1, GenBank: NM_001178065.2; 1,088 amino acid residues) in genome build GRCh37.p13. Nonsense and frameshift variants were assessed for nonsense-mediated decay by *in silico* analysis (NMDEscpredictor). We used a stepwise approach to identify all possible impactful missense variants. Initial triage focused on all rare genetic variants and their predicted pathogenicity as determined by the bioinformatics pipeline (RMPath, Supplemental Methods), designated with terms consistent with American College of Medical Genetics and Genomics (ACMG) guildelines.^19^ The second step in triage extracted and analyzed all outpatient serum Ca measures (or median serum Ca_alb_ when available) for each individual. The final step in variant triage examined individual patient EHR to assess potential comorbidities that might impact serum Ca measurements (bariatric surgery, malabsorption syndromes, osteoporosis, and/or cancer). In cases where individuals presented with potential serum Ca modifying comorbidities, we plotted the timeline of serum Ca values to determine whether there were non-random trends. Variants present in one or more individuals were examined for familial associations.

### Prevalence/Penetrance Estimates for FHH1 and ADH1

Prevalence of FHH1 or ADH1 in unrelated individuals of the cohort was determined after removal of one individual of each related pair of carriers of LoF (denoting frameshift or nonsense variants) and mLoF (denoting missense loss of function) variants (FHH1) or mGoF (denoting missense gain of function) variants (ADH1). Familial penetrance was determined as the fraction of related carriers with the serum Ca phenotypes (mean serum Ca ≥ 10.2 mg/dL for FHH1; mean serum Ca ≤ 8.5 mg/dL for ADH1) normalized to total related carriers.

### Functional Characterization of Rare Variants

#### Expression and Plasma Membrane Targeting

Point mutations, generated by polymerase chain reaction in the most prevalent human CaSR transcript (GenBank: NM_000388.4, 1,078 amino acid residues; missing amino acid residues 537–547 of the longest predicted CaSR transcript [GenBank: NM_001178065.2]) in a FLAG-tagged CaSR cDNA (FLAG-CaSR in pcDNA3.1), were confirmed by sequencing (Genewiz) and transiently transfected into HEK293 cells (FuGENE HD, Promega). Plasma membrane targeting was determined by enzyme-linked immuno-absorbance assays (ELISA) with HRP-conjugated anti-FLAG antibody (Sigma-Aldrich) as previously described.^20^ ELISA detects the N-terminally expressed FLAG tag on the CaSR constructs and does not detect a specific CaSR epitope. Protein expression and processing to the mature form was assessed on western blots of lysates probed with a custom rabbit anti-CaSR antibody generated against the LRG extracellular epitope (amino acid residues 374–391),^20^ a region that did not encompass any of the studied variants.

#### Intracellular Calcium (Ca^2+^_i_) Measurements

We chose to functionally characterize CaSR variants by measuring extracellular Ca^2+^ (Ca^2+^_e_)-evoked changes in Ca^2+^_i_, since this is the pathway to which CaSR preferentially couples and is most commonly used for human variant analysis.^2^ Ca^2+^_i_ responses induced by graded increases in Ca^2+^_e_ in cells loaded with the Ca^2+^ indicator dye Fluo-4 were measured with a PHERAstar instrument (BMG Labtech) at 37°C, as previously described (Supplemental Methods).^21^ Assays were performed in four biological replicates in HEK293 cells transiently transfected with WT (= reference sequence) or CaSR variant cDNAs. The *F*-test was used for statistical analyses.^9^^,^^21^

## Results

### Identification and Triage of Rare *CASR* Variants

**Figure 1:**
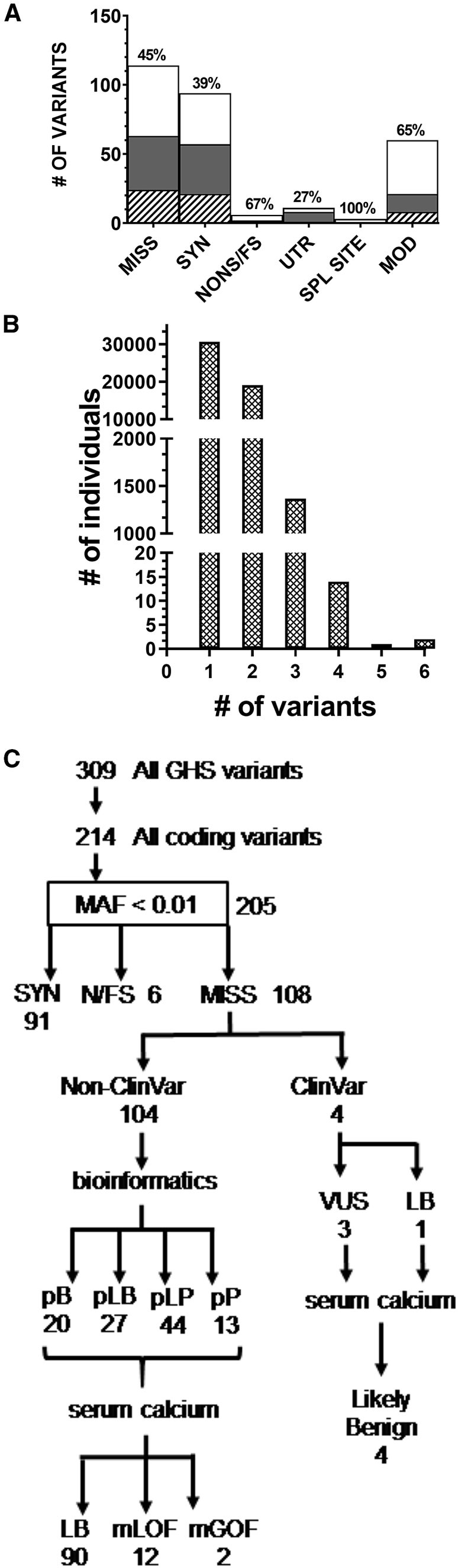
Characteristics and Bioinformatics Triage of *CASR* Variants in the 51,289 DiscovEHR Cohort (A) Variants were classified into coding (missense [MISS], synonymous [SYN], and nonsense or frameshift [NONS/FS]) and non-coding (3′ or 5′ untranslated region [UTR], splice site [SPL SITE], or upstream/downstream modifier [MOD]) variants. Bar graph indicates numbers of variants in each class. The percent of variants not previously listed in variant databases is indicated above each bar. Filled or shaded portions of bars indicate the subset of variants new to this study (white), with RefSeq rsID number (gray) or without ID but found in population variant databases (crosshatched). (B) Histogram of the common plus rare variant distribution in the 51,289 individual DiscovEHR cohort: 30,676 individuals had a single variant and 20,514 individuals had two or more variants (common + rare). (C) Rare missense variants (n = 108) were sorted into those with (n = 4) and without (n = 104) ClinVar annotation. Variants were triaged by RMPath (see Supplemental Subjects and Methods) from variants of unknown significance into those predicted to be benign (pB), likely benign (pLB), likely pathogenic (pLP), and pathogenic (pP). Analysis of serum Ca concentrations allowed further sorting of all heterozygous missense variants into likely benign (LB), missense loss-of-function (mLoF), and missense gain-of-function (mGoF) categories. MAF, minor allele frequency; SYN, synonymous variant; N/FS, nonsense or frameshift variant; MISS, missense variant; VUS, variant of unknown significance.

*CASR* variants in the 51,289 individual DiscovEHR cohort were identified and classified as coding (missense, synonymous, nonsense, or frameshift) or non-coding (3′ or 5′ UTR, splice site, or downstream/intronic or modifier) variants (Figure 1A). Sixty percent (30,676 of 51,289 individuals) had at least one common or rare *CASR* variant which might impact serum calcium (309 total variants, Figure 1B). A total of 205 different rare (mean allele frequency [MAF] < 0.01) coding variants were identified in 883 individuals, including 92 variants that were not found in public databases (dbSNP, ExAC [now in gnomAD], 1000 Genomes, or ESP). There were no significant domain biases in variant localization (Figure S1). Rare synonymous (91) and potential splice site variants (3) were not further pursued. We identified 6 nonsense/frameshift variants in 20 individuals. To assess the clinical impact of the rare missense variants, a two-tier triage based on bioinformatics assessment and measured serum Ca concentrations was undertaken (Figure 1C). Of the 57 variants predicted to be likely pathogenic (pLP) or pathogenic (pP), 12 were associated with hypercalcemia in 23 individuals, and 2 hypocalcemia-associated variants were identified in 3 individuals (Figure S2). Thus bioinformatics reduced the query set to 55% of total rare variants (57 LP/P variants from a total of 104), and subsequent serum Ca concentration analysis identified the 25% of total rare variants that were potentially associated with FHH1 or ADH1 phenotypes. None of the variants predicted to be likely benign (pLB) or benign (pB) had individuals with serum Ca concentrations at the outer limits of the normal range, and they were not further investigated.

### Serum Ca Concentrations of Carriers of *CASR* Nonsense, Frameshift, or Missense Variants

**Figure 2:**
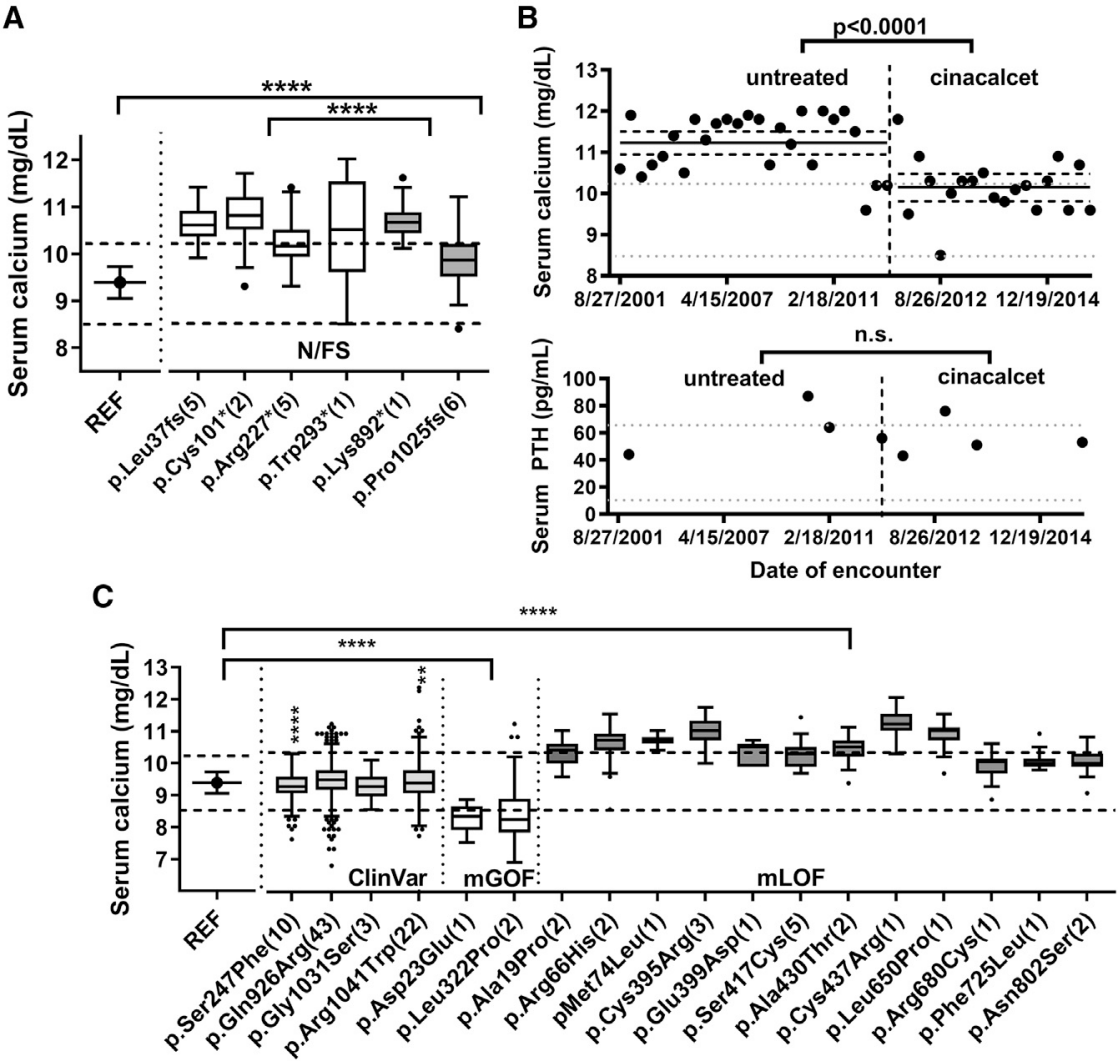
Serum Ca Concentrations for Individuals Having LoF or Rare Missense Variants in the DiscovEHR Cohort (A) Box and whisker plot in the style of Tukey (points indicating outliers) of all serum Ca measures from the EHR summed for all individuals having the same nonsense or frameshift variant (# of individuals). White boxes indicate variants predicted to undergo NMD, gray boxes indicate variants predicted to escape NMD, and dotted lines mark the normal serum Ca concentration range from 8.5 to 10.2 mg/dL. Also plotted is the mean ± SD of all individuals (15,922) having no rare variants but homozygous for the reference (REF) haplotype (Ala986/Arg990/Glu1011, residue numbering corresponding to GenBank: NM_000388.4). ^∗∗∗∗^p < 0.0001. (B) Upper plot presents the time series of serum Ca measures for an individual with a heterozygous Trp293^∗^ variant, showing a significant decrease in mean total serum Ca concentrations in December 2011, when treatment with cinacalcet began, as indicated by vertical dotted line (p < 0.0001). Means and 95% confidence intervals are plotted (mean prior to cinacalcet initiation, 11.23 ± 0.69 mg/dL, 95% C.I., 10.95–11.5 mg/dL; after cinacalcet initiation, 10.15 ± 0.69 mg/dL, 95% C.I., 9.81–10.48 mg/dL). Cinacalcet was continued through the beginning of 2015 and had a statistically significant effect on serum Ca concentrations (p < 0.0001). Lower plot indicates serum PTH values determined before and after cinacalcet initiation; differences in mean values were not significant. (C) Serum Ca means (box and whiskers in style of Tukey with points indicating outliers) were plotted for individuals homozygous for the REF haplotype (15,922 individuals), and carriers of ClinVar and rare missense *CASR* variants that affect serum Ca concentrations (residue numbering according to GenBank: NM_000388.4). The number of carriers of each variant having serum Ca measures in the EHR are noted in parentheses. Dotted lines mark the normal range for serum calcium (8.5–10.2 mg/dL). Statistically significant differences between groups was determined by ANOVA (^∗∗^p < 0.01, ^∗∗∗∗^p < 0.0001).

Six nonsense or frameshift variants were identified (= N/FS, Figure 1C; [transcript 1, GenBank: NM_001178065.2]). The four variants that were localized in the ECD (c.108dup [p.Leu37Alafs^∗^11], c.303C>A [p.Cys101^∗^], c.679C>T [p.Arg227^∗^], c.879G>A [p.Trp293^∗^]) were predicted to undergo nonsense-mediated mRNA decay (NMD), while two variants in the CaSR CT (c.2674A>T [p.Lys892^∗^], c.3074delC [p.Pro1025Argfs^∗^9]) escape NMD and are predicted to result in truncated receptors. The variants subject to NMD were identified in 13 individuals and had an aggregate mean serum Ca concentration significantly higher than the 7 individuals with non-NMD variants (NMD, 10.57 ± 0.63 [n = 268 values] versus non-NMD, 10.03 ± 0.56 [n = 86 measures], p < 0.0001 by two-tailed t test). Individuals with either class of nonsense/frameshift variant had mean serum Ca concentrations significantly higher than individuals having no rare variants and homozygous for the reference (REF) haplotype (corresponding to Ala986/Arg990/Glu101; residue numbering based on GenBank: NM_000388.4) (Ca 9.39 ± 0.34, n = 15,922, p < 0.0001 by one-way ANOVA) (Figure 2A and Table 1). Available EHR data confirm that most hypercalcemic individuals heterozygous for nonsense/frameshift variants had normal or slightly elevated serum PTH levels, and urinary Ca levels below the normal range, consistent with the FHH1 phenotype (Table 1). The individual heterozygous for c.879G>A (p.Trp293^∗^) had longitudinal EHR data that documented the effect of the allosteric activator of CaSR, cinacalcet (Sensipar), on serum Ca concentrations. Serum Ca was consistently elevated (10.2–12 mg/dL) prior to treatment (all measures plotted in Figure 2B), then decreased into the high-normal range after initiation of treatment. Cinacalcet caused a reduction in serum PTH concentrations into the normal range, although the differences were not statistically significant, likely due to the low number of measures under each condition (73.0 ± 12.4 pg/mL, n = 5, before treatment; 63.8 ± 9.7 pg/mL, n = 5, on cinacalcet; p = 0.23) (Figure 2B). The two urinary Ca measures available in the EHR were both acquired prior to initiation of cinacalcet treatment and were uninformative as to the effect of cinacalcet (Table 1). Overall, results confirm that CaSR nonsense/frameshift variants promote hypercalcemia.

**Table 1:** Demographics, Laboratory Values, and Variant Burden of Heterozygous Carriers of *CASR* Nonsense and Frameshift Variants

**Sex**	**Age (year)**	**Mean Serum Ca mg/dL**^a^**(# measures) [range]**	**Median Serum Ca**_**alb**_**mg/dL**	**Mean Urinary Ca g**^b^**(# measures)**		**PTH pg/mL**^c^**(# measures) [range]**
**c.108dup (p.Leu37Alafs^∗^11)**						

F	49	10.6 ± 0.4 (16) [9.9–10.7]	10.5		0.14 ± 0.029 (3)	46.5 ± 14.3 (8) [24–71]
M	74	10.7 ± 0.4 (23) [9.9–11.4]	10.7		N/A	127.5 ± 48 (6) [74–208]
M	72	10.8 ± 0.3 (7) [8.8–11.2]	10.7		N/A	33(1)
F	61	10.5 ± 0.4 (12) [10.3–11.0]	10.4		0.023 (1)	129.8 ± 0.43 (8) [66–209]
M	44	10.3 ± 0.2 (3) [10.0–10.4]	10.4		N/A	N/A

**c.303C>A (p.Cys101^∗^)**						

F	33	10.2 ± 0.4 (7) [9.3–10.4]	N/A		N/A	N/A
M	83	10.5 ± 0.7 (164) [9.7–11.7]	10.5		0.171 (1)	53.7 ± 30 (14) [26–124]

**c.679C>T (p.Arg227^∗^)**						

F	65	10.5 ± 0.4 (28) [9.9–11.3]	10.2		0.057 (1)	73.6 ± 16 (5) [56–90]
F	73	10.6 ± 0.2 (3) [10.5–10.8]	9.5		0.049 (1)	45.6 ± 8 (3) [38–54]
M	45	10.0 ± 0.4 (5) [9.4–10.2]	N/A		N/A	45 (1)
M	68	10.0 ± 0.4 (24) [9.5–10.5]	9.72		N/A	146.5 ± 13.4 (2) [137–156]
F	32	9.9 (1)	N/A		N/A	N/A

**c.879G>A (p.Trp293^∗^)**						

F	77	10.5 ± 1.0 (54) [8.5–12.0]	10.01		0.133 ± 0.04 (2)	64.7 ± 21 (12) [40–102]

**c.2674A>T (p.Lys892^∗^)**						

F	26	10.7 ± 0.4 (16) [10.1–11.6]	10.37		N/A	51.3 ± 11 (9) [39–75]

**c.3074delC (p.Pro1025Argfs^∗^9)**						

M	90	8.4 ± 0.4 (4) [8.4–9.4]	N/A		N/A	N/A
F	64	9.7 ± 0.3 (9) [9.2–10.2]	N/A		N/A	N/A
F	74	10.1 ± 0.3 (7) [9.5–10.7]	10.1		N/A	42.8 ± 9.8 (5) [33–54]
F	81	10.3 ± 0.5 (7) [9.7–11.2]	N/A		N/A	41 (1)
F	81	9.5 ± 0.4 (16) [8.9–10.2]	N/A		N/A	56 (1)
M	25	9.4 (1)	N/A		N/A	N/A

We present both mean serum Ca, and when available, albumin-corrected median serum Ca values, urinary Ca, and serum PTH values. N/A, no measures available in the EHR. Only the individual with the p.Trp293^∗^ variant was treated with cinacalcet; no individuals were treated with vitamin D during the time period of serum Ca measures. Genomic variants are designated relative to the *CASR* reference sequence (GenBank: NM_001178065.2).

a Normal range for serum Ca is 8.5–10.2 mg/dL.

b Normal range for urinary Ca is 0.1–0.25 g/24 h.

c Reference range for intact serum PTH is 15–65 pg/mL.

Four variants in the cohort were found in ClinVar (Clinical Variant database), an NCBI archive of medically important human variation, and were scored as variants of unknown significance (VUS) (c.740C>T [p.Ser247Phe], c.2807A>G [p.Gln936Arg], c.3151C>T [p.Arg1051Trp]) or benign/likely benign (c.3121G>A [p.Gly1041Ser]) (transcript 1, GenBank: NM_001178065.2). Despite the means of all individual carriers for each ClinVar variant being in the middle quartiles of the normal serum Ca concentration range (8.925–9.775 mg/dL), individuals with p.Ser247Phe and p.Arg1041Trp variants had significantly lower mean serum Ca concentrations than individuals homozygous for the reference *CASR* haplotype (p.Ser247Phe, 9.21 ± 0.44 mg/dL [10 individuals], p < 0.0001 versus REF; p.Arg1051Trp, 9.33 ± 0.59 mg/dL [22 individuals], p = 0.005 versus REF; REF 9.39 ± 0.38 mg/dL [15,922 individuals]). However, neither p.Gln936Arg (9.38 ± 0.55 mg/dL, 43 individuals) nor p.Gly1041Ser (9.2 ± 0.42 mg/dL, 3 individuals) significantly altered mean serum Ca concentrations relative to *CASR* REF. Similar analyses were done on the 14 pLP and pP variants having serum Ca concentration distributions at the outer limits of the normal range. We identified 13 hypercalcemic individuals (mean serum Ca concentrations ≥ 10.2 mg/dL) who were carriers of 9 distinct rare variants consistent with missense loss-of-function (mLoF) variants, and 3 variants in 8 individuals were associated with serum Ca concentrations that were significantly elevated relative to the *CASR* REF haplotype (p < 0.0001), i.e., in the upper quartile of normal (9.775–10.2 mg/dL). Two missense variants were associated with low serum Ca concentrations (means ≤ 8.5 mg/dL) in 3 individuals, consistent with missense gain-of-function (mGoF) mutations (Figure S2, clinical data in Table 2).

**Table 2:** Demographics, Laboratory Values, and Variant Burden of Heterozygous Carriers of Rare *CASR* Missense Variants

**Sex**	**Age (year)**	**Mean Serum Ca mg/dL**^a^**(# measures) [range]**	**Median Serum Ca**_**alb**_**mg/dL**	**Mean Urinary Ca g**^b^**(# measures)**	**PTH pg/mL**^c^**(# measures) [range]**
**c.69C>A (p.Asp23Glu)**					

M	64	8.25 ± 0.4 (18) [7.5–8.8]	8.16	N/A	N/A

**c.965T>C (p.Leu322Pro)**					

F	55	7.8 ± 0.6 (12) [6.9–8.5]	7.49	N/A	28.8
M	79	8.6 ± 0.9 (54) [7.5–9.1]	7.5	N/A	14.7 ± 6.6 (7) [7–26]

**c.55G>C (p.Ala19Pro)**					

M	69	10.23 ± 0.4 (10) [9.5–10.6]	N/A	N/A	N/A
F	35	10.3 (1)	N/A	N/A	N/A

**c.197G>A (p.Arg66His)**					

M	82	10.5 ± 0.5 (80) [8.5–11.4]	10.62	N/A	94 (1)
F	60	10.43 ± 0.3 (5) [10.3–10.7]	N/A	N/A	N/A

**c.220A>C (p.Met74Leu)**					

M	63	10.6 ± 0.4 (2) [10.3–10.9]	N/A	N/A	N/A

**c.1183T>C (p.Cys395Arg)**					

F	65	10.8 ± 0.4 (16) [9.9–11.6]	10.64	0.100 (1)	49.5 ± 16.5 (11) [21–77]
F	40	10.94 ± 0.5 (9) [10.1–11.3]	N/A	0.12 ± 0.019 (2)	29 ± 7.4 (9) [18–43]
M	44	10.9 ± 0.4 (13) [10.3–11.5]	10.25	N/A	34 (1)

**c.1197G>C (p.Glu399Asp)**					

F	55	10.2 ± 0.4 (5) [9.8–10.6]	N/A	N/A	N/A

**c.1250C>G (p.Ser417Cys)**					

M	55	10.1 ± 0.3 (24) [9.6–10.8]	9.8	N/A	12.5 ± 3.5 (2) [10–15]
F	23	N/A	N/A	N/A	N/A
M	65	10.4 ± 0.6 (5) [9.8–11.3]	N/A	N/A	N/A
F	70	9.87 ± 0.3 (3) [9.7–10.2]	N/A	N/A	70.5 ± 23.3 (2) [54–87]
F	42	10.1 ± 0.3 (3) [9.8–10.4]	N/A	N/A	N/A

**c.1288G>A (p.Ala430Thr)**					

F	70	10.1 ± 0.3 (16) [9.7–11.0]	9.8	N/A	N/A
M	72	10.4 ± 0.3 (65) [9.7–11.0]	10.28	N/A	33 (1)

**c.1309T>C (p.Cys437Arg)**					

M	85	11.1 ± 0.4 (46) [10.2–11.9]	11.18	0.131 ± 0.01 (2)	33.5 ± 10.8 (4) [18–41]
F	73	9.4 ± 0.3 (6) [9.0–9.7]	N/A	N/A	127 (1)

**c.1979T>C (p.Leu660Pro)**					

F	65	10.8 ± 0.4 (27) [9.6–11.4]	10.76	0.06 ± 0.03 (2)	43.7 ± 7.8 (9) [33–53]

**c.2068C>T (p.Arg690Cys)**					

F	76	9.86 ± 0.4 (27) [8.8–10.5]	9.65	N/A	N/A

**c.2205C>G (p.Phe735Leu)**					

F	69	10.0 ± 0.27 (17) [9.7–10.8]	9.68	N/A	N/A

**c.2435A>G (p.Asn812Ser)**					

F	64	9.9 ± 0.4 (54) [9–10.7]	9.12	N/A	N/A
M	43	10.0 ± 0.3 (25) [9.5–10.6]	9.58	N/A	N/A

We present both mean serum Ca, and when available, albumin-corrected median serum Ca values, urinary Ca, and serum PTH values. N/A, no measures available in the EHR. No individuals were treated with cinacalcet or vitamin D during the time period of serum Ca measures. Genomic variants are designated relative to the *CASR* reference sequence (GenBank: NM_001178065.2).

a Normal range for serum Ca is 8.5–10.2 mg/dL.

b Normal range for urinary Ca is 0.1–0.25 g/24 h.

c Reference range for intact serum PTH is 15–65 pg/mL.

In 41,489 individuals not treated with cinacalcet or vitamin D, we identified 333 individuals (0.78%) with overt hypercalcemia (≥10.2 mg/dL, range 10.2 to 12.8 mg/dL); the 25 individuals with rare LoF or mLoF *CASR* variants having mean serum Ca concentrations ≥ 10.2 mg/dL represented 7.5% of overtly hypercalcemic individuals. There were 1,699 individuals with hypocalcemia (mean serum Ca concentration ≤ 8.5 mg/dL, range 6.3 to 8.5 mg/dL) who were not taking cinacalcet or vitamin D; the 3 individuals with rare *CASR* mGOF variants represent 0.18% of hypocalcemic individuals.

### Concordance of FHH1 and ADH1 Phenotypes in Related Individuals

Table 3 illustrates the results for carriers of LoF (9 kindreds, 4 variants) and missense (11 kindreds, 9 variants) variants that demonstrate familial hyper- or hypocalcemia. Available clinical values of related individuals support the causal impact of *CASR* haplo-insufficiency in elevated mean/median serum Ca concentrations (Table 3). As suggested by the aggregate data in Figure 2A, individuals with the p.Pro1025Argfs^∗^9 variant were at the high end of normal serum Ca concentrations and relations without the variant consistently had lower serum Ca concentrations. A similar pattern of autosomal-dominant inheritance of serum Ca concentrations was observed for carriers of *CASR* missense variants (Table 3). Overall, relatedness analysis was consistent with autosomal-dominant inheritance of serum Ca concentrations in heterozygous carriers of *CASR* variants.

**Table 3:** Relatedness of Heterozygous Carriers of Rare Nonsense, Frameshift, and Missense Variants in *CASR,* and Serum Ca, Albumin-Corrected Serum Ca Values, Urinary Ca, and Serum for Heterozygous Individuals in the 92K DiscovEHR Cohort

**Variant**	**Age (year)**	**Sex**	**Mean Serum Ca mg/dL**^a^**(# measures)**	**Median Serum Ca**_**alb**_**mg/dL**	**Mean Urinary Ca g**^b^**(# measures)**	**PTH pg/mL**^c^**(# measures)**	**Relatedness**^d^	**Pi-HAT**
**Nonsense/Frameshift Variants**								

p.Leu37Alafs^∗^11	44	M	10.3 ± 0.23 (3)	10.4	N/A	N/A	proband^∗^	0.5
p.Leu37Alafs^∗^11	61	F	10.5 ± 0.35 (12)	10.4	0.23(1)	139.4 (8)	parent^∗^	
p.Leu37Alafs^∗^11	74	M	10.7 ± 0.4 (23)	10.7	N/A	127.4 ± 48 (6)	proband^∗^	0.5304
none	77	F	9.93 ± 0.37 (28)	10.0	N/A	47.5 ± 9.3 (4)	full sibling	
p.Cys101^∗^	33	F	10.2 ± 0.4 (7)	10.4	N/A	N/A	proband^∗^	0.2537
p.Cys101^∗^	83	M	10.9 ± 0.4 (28)	10.9	0.171 (1)	47.6 ± 27 (11)	2°^∗^	
p.Arg227^∗^	65	F	10.5 ± 0.44 (28)	10.4	0.057 (1)	73.6 ± 16 (5)	proband^∗^	
none	90	F	9.83 ± 0.4 (53)	9.9	N/A	N/A	parent	0.5
none	47	M	9.7 ± 0.3 (8)	9.6	N/A	N/A	child	0.5
none	71	F	9.6 ± 0.4 (28)	9.5	N/A	N/A	full sibling	0.5578
p.Arg227^∗^	45	M	10.13 ± 0.4 (8)	10.2	N/A	45 (1)	proband^∗^	0.2513
none	55	M	9.49 ± 0.53 (19)	9.6	N/A	18 (1)	2°	
p.Arg227^∗^	68	M	9.95 ± 0.4 (24)	10	N/A	146.5 ± 13 (2)	proband^∗^	0.5112
none	63	F	9.32 ± 0.2 (11)	9.3	N/A	41.5 ± 21.5 (2)	full sibling	
p.Pro1025Argfs^∗^9	64	F	9.67 ± 0.34 (9)	9.7	N/A	N/A	proband^∗^	
none	81	F	9.64 ± 0.3 (12)	9	N/A	N/A	parent	0.5142
none	84	M	9.22 ± 0.3 (32)	9.2	N/A	N/A	2°	0.2413
none	32	F	9.3 ± 0.35 (3)	9.1	N/A	N/A	2°	0.2889
p.Pro1025Argfs^∗^9	74	F	10.1 ± 0.3 (27)	10.2	N/A	42.8 ± 9.8 (4)	proband^∗^	
none	27	M	9.7 (1)	9.7	N/A	N/A	2°	0.299
none	29	M	9.68 ± 0.39 (12)	9.5	N/A	N/A	2°	0.2142
p.Pro1025Argfs^∗^9	81	F	9.64 ± 0.3 (25)	9.6	N/A	56 (1)	proband^∗^	
none	84	M	9.22 ± 0.3 (32)	0.2	N/A	N/A	full sibling	0.4886
none	M	63	9.28 ± 0.35 (5)	9.1	N/A	47.3 ± 16 (4)	2°	0.2455
none	32	F	9.3 ± 0.4 (3)	9.1	N/A	N/A	2°	0.2917

**Missense Variants**								

p.Asp23Glu	64	M	8.25 ± 0.4 (18)	8.3	N/A	N/A	proband^∗^	0.5787
none	69	M	9.0 ± 0.3 (9)	9	N/A	N/A	full sibling	
p.Leu322Pro	56	F	8.39 ± 0.4 (154)	8.4	N/A	30.2 ± 13 (10)	proband^∗^	
p.Leu322Pro	79	M	8.6 ± 0.9 (54)	8.3	N/A	14 ± 6.1 (9)	parent^∗^	0.4946
none	77	F	9.95 ± 0.5 (29)	10	N/A	33 (1)	parent	0.4977
p.Ala19Pro	69	M	10.23 ± 0.41 (10)	10.2	N/A	N/A	proband^∗^	0.5093
p.Ala19Pro	40	F	10.2 ± 0.3 (8)	10.1	N/A	N/A	child^∗^	
pAla19Pro	35	F	10.3	10.3	N/A	N/A	proband^∗^	0.1918
none	79	M	9.01 ± 0.34 (10)	9	N/A	N/A	2°	
p.Arg66His	60	F	10.44 ± 0.3 (4)	10.5	N/A	N/A	proband^∗^	0.2425
p.Arg66His	70	M	10 ± 0.3 (61)	10	N/A	N/A	2°^∗^	
p.Cys395Arg	65	F	10.84 ± 0.4 (16)	10.9	0.1 (1)	49.5 ± 17 (11)	proband^∗^	0.5
p.Cys395Arg	40	F	10.94 ± 0.5 (9)	10.9	0.13 ± 0.02 (2)	29 ± 7.4 (9)	child^∗^	
none	60	M	9.4 ± 0.24 (4)	9.2	N/A	N/A	2°	0.1898
p.Glu399Asp	55	F	10.2 ± 0.4 (5)	10.4	N/A	N/A	proband^∗^	0.1893
none	M	65	9.2 ± 0.24 (6)	9.2	N/A	N/A	2°	
p.Ser417Cys	65	M	10.4 ± 0.6(5)	10.3	N/A	N/A	proband^∗^	0.5037
none	66	F	9.29 ± 0.33 (9)	9.3	N/A	N/A	full sibling	
p.Ser417Cys	55	M	10.13 ± 0.33 (24)	10.1	N/A	12.5 ± 3.4 (2)	proband^∗^	
p.Ser417Cys	90	F	10.1 ± 0.3 (18)	10.1	N/A	40 (1)	2°^∗^	0.298
none	74	M	9.18 ± 0.23 (11)	9.2	N/A	N/A	full sibling	0.5272
p.Leu660Pro	45	F	10.5 (1)	10.5	N/A	N/A	proband^∗^	0.2201
p.Leu660Pro	65	F	10.9 ± 0.4 (27)	10.9	0.06 ± 0.03 (2)	43.7 ± 7.8 (9)	2°^∗^	
p.Arg690Cys	76	F	9.87 ± 0.4 (27)	10	N/A	N/A	proband^∗^	0.3389
none	55	M	9.14 ± 0.44 (4)	9.1	N/A	N/A	2°	

N/A, no measures in EHR. Parent-Child (P-C), full sibling, or 2° (unspecified) relations are listed. Genomic variants are designated relative to the *CASR* reference sequence (GenBank: NM_001178065.2).

a Normal range for serum Ca is 8.5–10.2 mg/dL.

b Normal range for urinary Ca is 0.1–0.25 g/24 h.

c Reference range for serum PTH is 10–65 pg/mL.

d Asterisk indicates variant carrier.

### Functional Characterization of the CaSR mGoF/mLoF Variants

The means of all serum Ca measures combined for all individuals heterozygous for each ClinVar and potential FHH1 or ADH1 missense variants are plotted in Figure 2C. Individuals with putative mGoF variants had significantly reduced mean serum Ca concentrations while all individuals having mLoF variants had mean serum Ca concentrations significantly higher than the REF (= ARE) haplotype and 9 of 12 variants had individuals who were overtly hypercalcemic (≥10.2 mg/dL) (Figure 2C). To validate pathogenicity predictions for the putative mGoF and mLoF variants, we introduced each into a human FLAG-tagged CaSR (based on transcript GenBank: NM_000388.4) construct by site-directed mutagenesis, and transiently transfected wild-type (WT = REF) or mutant constructs into HEK293 cells, as described.^20^

**Figure 3:**
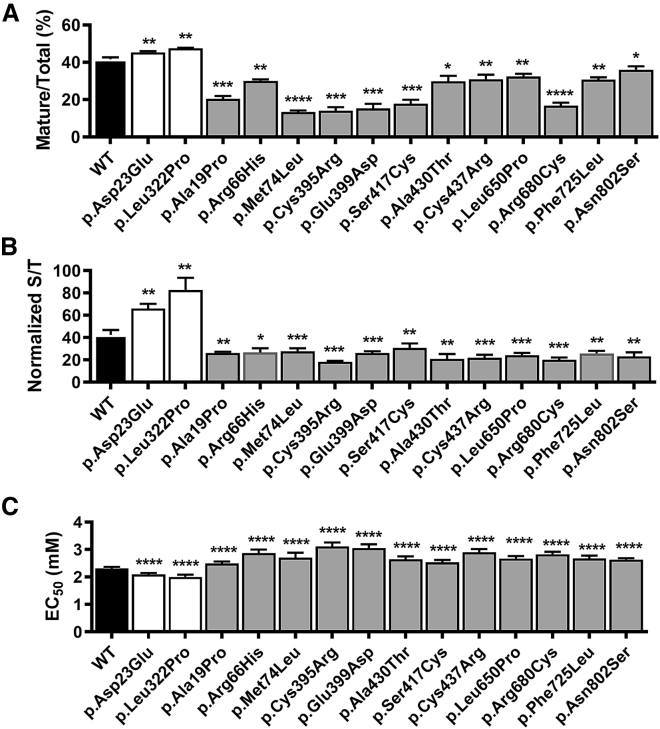
Biochemical and Cellular Analysis of mLoF and mGoF CaSR Mutations (A) Plot presents mean ± SD of western blot analysis of lysates from three independent HEK293 cell transfections, as the percent of mature receptors ([mature/(immature + mature)]%) for WT (black bar), mGoF (white bars), and mLoF (gray bars) variants. Representative western blots presented in Figure S3. Statistical analysis was by Student’s t test, with ^∗^p < 0.05, ^∗∗^p < 0.01, ^∗∗∗^p < 0.001, and ^∗∗∗∗^p < 0.0001. (B) Plasma membrane localization was determined for WT (black bar), mGoF (white bars), and mLoF (gray bars) variants by ELISA of transiently transfected HEK293 cells, plotted as the mean ± SD of the percent of surface to total expression, for at least three independent experiments. Statistical analysis was by Student’s t test, with ^∗^p < 0.05, ^∗∗^p < 0.01, ^∗∗∗^p < 0.001, and ^∗∗∗∗^p < 0.0001. (C) Bar graph of EC_50_ values for WT (black bar), mGoF (white bars), and mLoF (gray bars) variants extracted from fits of dose-response relations for Ca^2+^_e_. Statistical analysis by *F*-test, with ^∗∗∗∗^p < 0.0001. Titrations were done as described in Subjects and Methods, and plots of dose-response relations which were fitted to extract EC_50_s are presented in Figures S5 and S6. Numbering of residues for all graphs based on GenBank: NM_000388.4.

We compared protein levels and plasma membrane targeting of each mGoF and mLoF variant relative to WT by western blotting and ELISA.^20^ The antibodies used for these studies targeted either an inserted epitope (amino terminal FLAG, used for ELISA) or an endogenous ECD sequence (LRG epitope, residues 374–391, used for western blotting) that did not contain tested variants. The mGoF variants increased total and plasma membrane protein levels whereas mLoF variants reduced total and plasma membrane protein levels relative to WT CaSR, Figures 3A and 3B (representative western blot in Figure S3). To assess WT and variant function, we measured Ca^2+^_i_ mobilization in response to alterations in Ca^2+^_e_ concentrations in transiently transfected cells.^22^ Fitting of the Ca^2+^_e_ concentration-response curves (Figures S4-S5) showed that mGoF variants had significantly reduced EC_50_s, i.e., [Ca^2+^_e_] at half-maximal response, whereas the 12 mLoF variants had significantly increased EC_50_s relative to WT CaSR (Figure 3C). These combined studies demonstrate altered protein expression, plasma membrane localization, and/or activation by Ca^2+^_e_ consistent with predicted mGoF or mLoF variants, as revealed by effects on serum calcium concentrations.

### Prevalence Estimates for FHH1 and ADH1 in the DiscovEHR Cohort

**Figure 4:**
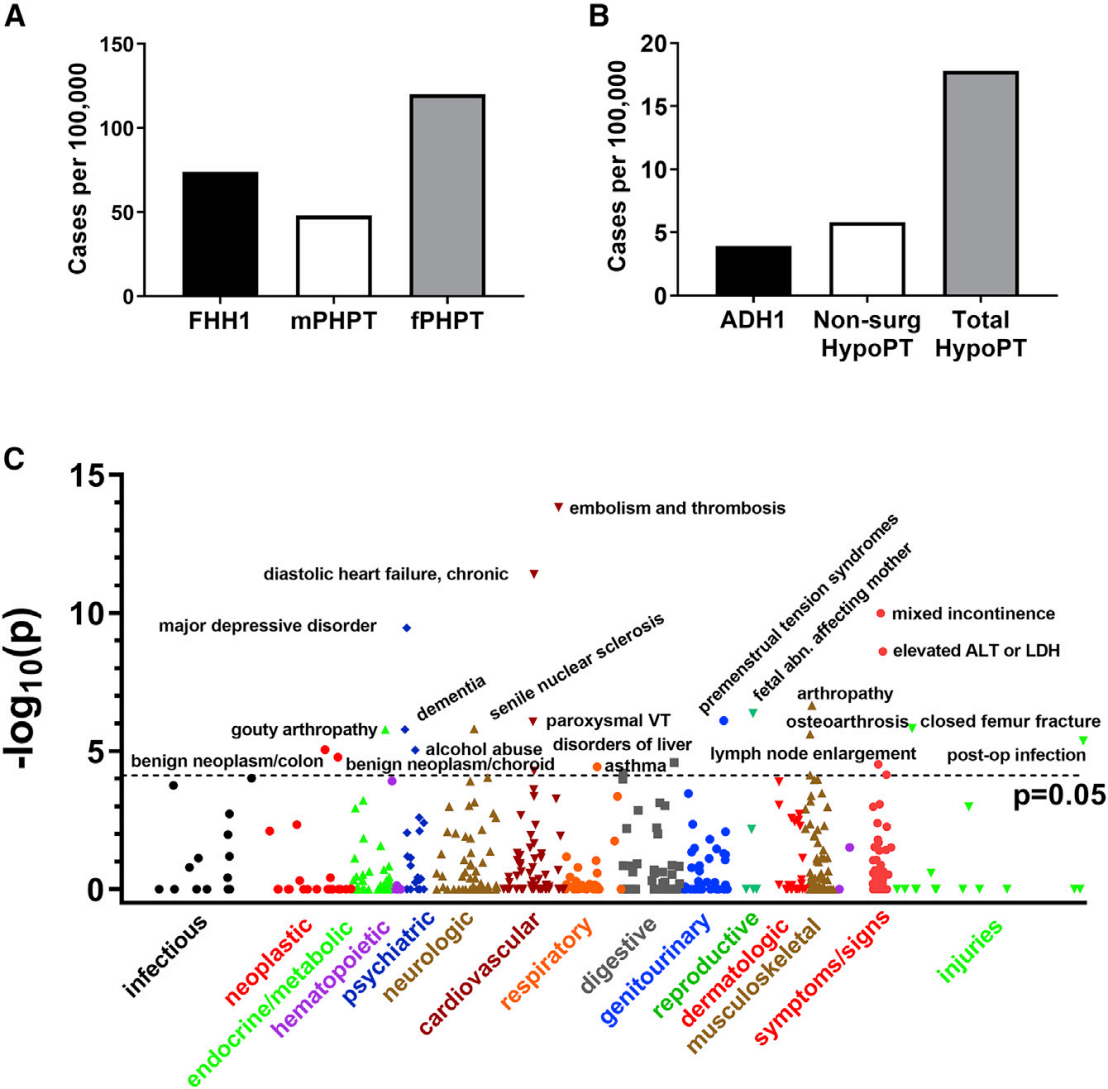
Prevalence of FHH1 and ADH1 in DiscovEHR Cohort (A) FHH1 prevalence in the DiscovEHR cohort (black bar) compared to the age-adjusted prevalence of male PHPT (mPHPT, white bar) and female PHPT (fPHPT, gray bar) cases reported in a large US cohort.^14^ (B) ADH1 prevalence in the DiscovEHR cohort (black bar) compared with prevalence of non-surgical hypoparathyroidism (non-surg HypoPT, white bar) and all forms of hypoparathyroidism (Total HypoPT, gray bar), estimated for the entire insured adult US population.^23^ (C) Manhattan plot of SKAT analysis used to detect disease associations defined by ICD9 codes for a binned group of all LoF plus mLoF variants. Labels indicate top associations with phenome-wide significance, which include all results above the dotted line, p < 0.05, corrected for multiple testing. Variants included in analysis and all significant results are in Table 4.

The clinical and functional data identify genetic variants that are associated with FHH1 or ADH1. We estimated the prevalence of these variants in the cohort and compared them with other know causes of hyper- or hypocalcemia (Figures 4A and 4B). Thirty-eight unrelated individuals (one of each related pair were removed from the analysis), yielded a prevalence of 74.1 per 100,000. Two unrelated individuals with ADH1 yielded a prevalence of 3.9 per 100,000. To estimate familial penetrance, i.e., the proportion of hypercalcemic relatives, we identified the subset of related individuals with genetic variants that expressed the serum Ca phenotype, i.e., for FHH1 the estimated familial penetrance was 92% (23 of 25 related individuals had mean serum Ca ≥ 10.2 mg/dL) and for ADH1, estimated familial penetrance was 100% (Tables 1 and 2).

### Association of FHH1 *CASR* Variants with Major Disease Phenotypes

We assessed whether genetically determined lifetime mildly elevated serum Ca concentrations and/or reduced functional activity of CaSR altered the risks of common non-calcitropic diseases. All individuals having *CASR* loss-of-function variants (nonsense plus mLoF missense variants), were combined for burden testing using the Sequence Kernel Association Test (SKAT). All significant associations are listed in Table 4. Top diseases associated with elevated serum Ca concentrations and/or reduced CaSR function include cardiovascular disease, including embolism and thrombosis, chronic diastolic heart failure, and paroxysmal ventricular tachycardia (Figure 4C, Table 4), and neurological disorders, including major depression, dementia, and alcohol abuse. In addition, *CASR* loss-of-function variants were associated with neck of femur fractures (Table 4), although the variants were not significantly associated with osteoporosis. The small number of individuals with mGoF variants precluded disease association analysis.

**Table 4:** Sequence Kernel Association Test (SKAT) of Binned Rare *CASR* Loss-of-Function Variants

**Code**	**Code Description**	**# Cases**	**p Value**	**Q-Value**
453.9	embolism and thrombosis of unspecified site	705	1.51E−14	9.26E−12
428.32	diastolic heart failure, chronic	467	4.1E−12	1.12E−9
788.33	mixed incontinence (male, female)	260	1.03E−10	1.59E−8
296.2	major depressive disorder, single episode, unspecified degree	248	3.56E−10	4.38E−8
790.4	nonspecific elevations of transaminase or lactic dehydrogenase	277	2.54E−9	2.6E−7
716.96	arthropathy unspecified, lower leg	306	2.26E−7	1.76E−5
427.1	paroxysmal ventricular tachycardia	471	8.81E−7	4.88E−5
820.8	fracture of unspecified part of neck of femur, closed	205	1.56E−6	6.71E−5
366.16	senile nuclear sclerosis	4,938	1.59E−6	6.76E−5
294.2	dementia, without behavioral disturbance	201	1.7E−6	6.98E−5
274	gouty arthropathy	349	1.71E−6	6.99E−5
715.15	osteoarthritis, localized, primary, pelvic region and thigh	293	2.48E−6	9.53E−5
998.59	other postoperative infection	423	4.42E−6	.00016
211.3	benign neoplasm of colon	3,176	8.88E−6	.00029
305	alcohol abuse, unspecified drinking behavior	223	9.24E−6	.0003
224.6	benign neoplasm of choroid	233	1.67E−5	.00051
573.8	other specified disorders of liver	205	2.59E−5	.00074
785.6	enlargement of lymph nodes	609	3.01E−5	.00083
493.91	asthma, unspecified type, with status asthmaticus	217	3.69E−5	.00097
428.22	systolic heart failure, chronic	472	5.37E−5	.0013
521.02	dental caries extending into dentine	248	6.13E−5	.0014
794.31	nonspecific abnormal electrocardiogram	339	7.13E−5	.0015
715.11	osteoarthrosis, localized, primary, involving shoulder region	229	7.48E−5	.0016

Rare nonsense/frameshift and validated missense loss-of-function variants were combined and association with all clinical phenotypes determined by SKAT. Dataset for analysis included 25 males (61 ± 16.2 years, 96% northern European descent) and 30 females (55.5 ± 19 years, 100% northern European descent) with variants compared against 30,448 individuals without rare *CASR* variants. Three calls of an ICD9 were required to define a case, and covariates were sex, age, age^2^, and first four PCs (see Subjects and Methods). We chose an exploratory cutoff of p < 0.05, significant results were at p < 7.74E−5, when corrected for multiple testing (646 ICD9 codes with ≥200 cases irrespective of genotype). Variants used for analysis were p.Ala19Pro (c.55G>C), p.Leu37Alafs^∗^11 (c.108dup), p.Arg66His (c.197G>A), p.Met74Leu (c.220A>C), p.Cys101^∗^ (c.303C>A), p.Arg227^∗^ (c.679C>T), p.Trp293^∗^ (c.879G>A), p.Cys395Arg (c.1183T>C), p.Glu399Asp (c.1197G>C), p.Ser417Cys (c.1250C>G), p.Ala430Thr (c.1288G>A), p.Cys437Arg (c.1309T>C), p.Leu660Pro (c.1979T>C), p.Arg690Cys (c.2068C>T), p.Phe735Leu (c.2205C>G), p.Asn812Ser (c.2435A>G), p.Lys892^∗^ (c.2674A>T), and p.Pro1025Argfs^∗^9 (c.3074delC) (all variants relative to transcript 1, GenBank: NM_001178065.2).

## Discussion

The availability of whole-exome sequence data from 51,289 individuals from a single health system coupled with phenotype-rich EHR data has provided a unique opportunity to determine the prevalence of FHH1- and ADH1-associated *CASR* variants. *CASR* missense loss- or gain-of-function variants are particularly difficult to identify with pathogenicity prediction tools alone.^24^ Our strategy was designed to overcome the main drawbacks of working with de-identified data that was not specifically obtained to diagnose FHH1 or ADH1 in small pedigrees, i.e., incomplete clinical data for definitive diagnosis. We combined WES, available clinical data, and statistical estimates of the relatedness of de-identified *CASR* variant carriers to other individuals in a larger sequenced population. The combined results support the expected autosomal-dominant inheritance of the FHH1 and ADH1 clinical phenotypes for the identified variants. Results were further validated by *in vitro* functional analyses. In 51,289 individuals, we identified 14 different rare missense variants which could be linked to FHH1 or ADH1. Our approach accurately identified previously known FHH1 variants in the cohort, including p.Cys395Arg^25^ and p.Arg680Cys.^26^

FHH1 is generally considered a rare disorder, i.e., a study of known FHH kindreds from the West of Scotland reported a minimum prevalence of 1.3 cases in 100,000,^13^ in the range of other monogenic hypercalcemic disorders including multiple endocrine neoplasia type 1 (MEN1) syndrome (3.3–10 cases per 100,000 [Orphanet database]). The DiscovEHR cohort genetic prevalence for FHH1, which includes normocalcemic individuals, is 74 cases per 100,000, of which 49/100,000 had the FHH1 phenotype (∼38-fold higher than previous estimates), in the range of the age-adjusted prevalence of classical PHPT of 48 per 100,000 in adult males^14^ and ∼120 per 100,000 for adult females^14^ (Figure 4A). In a recent study of individuals being evaluated for hypercalcemia, 6.1% of patients had rare *CASR* variants, 47% of which were not previously reported.^27^ Moreover, this study independently confirmed three of the FHH1-associated variants identified in the current study, p.Arg227^∗^, p.Ala430Thr, and p.Arg680Cys.^27^ The substantially higher prevalence of FHH1 when estimated at the population level highlights the importance of testing for FHH in all individuals with suspected PHPT, to avoid unnecessary parathyroidectomy.^6^^,^^28^^,^^29^ Finally, our study provides a prevalence estimate for ADH1 of 3.9 cases per 100,000, in the range of the estimated prevalence of 5.8 cases per 100,000 for non-surgical hypoparathyroidism in the adult U.S. population^23^ (Figure 4B). Thus, ADH1 likely represents a major cause of non-surgical hypoparathyroidism.

Our analysis suggests heterozygous nonsense/frameshift variants may be a more common cause of FHH1 than previously reported,^2^ i.e., 12 of 25 FHH1 individuals had nonsense/frameshift variants. Further, the presence of sufficient individuals with nonsense/frameshift variants that are subject to or escape from NMD-mediated mRNA decay provides preliminary evidence to suggest the conclusion that NMD-mediated haplo-insufficiency may produce a more severe Ca phenotype than generation of prematurely truncated receptors. FHH1 familial penetrance, i.e., proportion of hypercalcemic relatives, in this cohort (∼92%) was comparable to the >95% penetrance reported in FHH family studies,^30^ despite the limited size of most pedigrees and the potential range of comorbidities that may lower serum Ca concentrations, including renal impairment and/or vitamin D deficiency. For those singletons without related individuals in the cohort, a time-dependent mapping of individual clinical measures correlated with both contemporaneous ICD9 coding and the full range of prescription and non-prescription medications would provide a more comprehensive means of defining the impact of the variant on serum Ca concentrations. Nevertheless, our integrated approach has identified rare *CASR* variants causing FHH1 and ADH1 using the most readily available clinical data and without imposition of significant assumptions and can be readily generalizable to other clinical populations.

FHH1 is considered a benign disorder, limited to lifetime mildly elevated serum Ca concentrations. We took advantage of the sufficient numbers of individuals having nonsense/frameshift or missense LoF variants in the current cohort to determine whether lifetime elevated serum Ca concentrations and/or reduced CaSR activity are associated with altered risks of common (≥1:250) non-calcitropic diseases in the U.S. population.^18^^,^^31^ Strong associations were found with cardiovascular diseases, including embolism and thrombosis and diastolic heart failure, and with major depression (Figure 4C, Table 4). The associations identified by SKAT analysis do not provide directionality, but the risks of embolism/thrombosis and diastolic heart failure have been shown to be influenced by serum Ca concentrations;^32^^,^^33^ likewise, neuropsychiatric disorders have been associated with serum Ca concentrations in small case-control studies.^34^^,^^35^ CaSR is widely expressed in the cardiovascular^36^^,^^37^ and nervous^38^ systems, and it remains to be determined whether these disease associations result from elevated serum Ca concentrations, reduced CaSR function, or both.

Overall, the benefits of our study are the ability to consider a broad range of clinical conditions in an unbiased manner and to corroborate results with quantitative clinical measures. Limitations of the approach include use of de-identified genomic and clinical data, precluding individual contact for more extensive phenotyping, and lack of specialized clinical data and/or testing to support particular diagnoses. Nevertheless, the large numbers of individuals and extensive clinical measures allowed for statistically significant findings, which can be followed up with specifically recruited cohorts. Finally, replication of FHH1/ADH1 prevalence estimates and FHH1 disease associations should be possible as independent population sequencing efforts combining WES and EHR data come to fruition.

In conclusion, we used a DiscovEHR cohort to develop a triage method for rapid identification of FHH1 and ADH1 candidate variants, verified by expression and functional studies. We estimate the prevalence of FHH1 and ADH1 and reveal that FHH1 may be >60-fold more common than previously reported. As sequencing in clinical practice becomes more routine, identifying FHH1 and ADH1 individuals in large cohorts may improve clinical care of these individuals, particularly as we identify comorbidities that accompany serum Ca^2+^ dysregulation and/or altered CaSR function.

## Declaration of Interests

The authors declare no competing interests.

## Consortia

Regeneron Genetics Center members: Goncalo Abecasis, Xiaodong Bai, Suganthi Balasubramanian, Nilanjana Banerjee, Aris Baras, Christina Beechert, Andrew Blumenfeld, Michael Cantor, Giovanni Coppola, Yating Chai, Amy Damask, Colm O’Dushlaine, Aris Economides, Gisu Eom, Caitlin Forsythe, Jan Freudenberg, Erin D. Fuller, Claudia Gonzaga-Jauregui, Nehal Gosalia, Zhenhua Gu, Lauren Gurski, Paloma M. Guzzardo, Lukas Habegger, Young Hahn, Alicia Hawes, Julie Horowitz, Marcus B. Jones, Shareef Khalid, Michael Lattari, Alexander Li, Nan Lin, Daren Liu, Alexander Lopez, Kia Manoochehri, Jonathan Marchini, Anthony Marcketta, Evan K. Maxwell, Shane McCarthy, Lyndon J. Mitnaul, John D. Overton, Charles Paulding, John Penn, Kavita Praveen, Jeffrey G. Reid, Thomas D. Schleicher, Claudia Schurmann, Maria Sotiropoulos Padilla, Karina Toledo, Louis Widom, Sarah E. Wolf, Manasi Pradhan, Alan Shuldiner, Jeffrey C. Staples, Dylan Sun, Tanya Teslovich, Ricardo H. Ulloa, Cristopher Van Hout, Ashish Yadav, and Bin Ye.

## Web Resources

1000 Genomes, http://www.internationalgenome.org/
dbSNP, https://www.ncbi.nlm.nih.gov/projects/SNP/
DiscovEHR, http://www.discovehrshare.com/
gnomAD Browser, https://gnomad.broadinstitute.org/
NHLBI Exome Sequencing Project (ESP) Exome Variant Server, http://evs.gs.washington.edu/EVS/
NMDEscPredictor, https://nmdprediction.shinyapps.io/nmdescpredictor
OMIM, https://www.omim.org/

## Supplemental Data

Document S1. Figures S1–S5, Table S1, and Supplemental Methods

Document S2. Article plus Supplemental Information
