# Supplementary material for: Familial Hypocalciuric Hypercalcemia Type 1 and Autosomal-Dominant Hypocalcemia Type 1: Prevalence in a Large Healthcare Population
Source: Am J Hum Genet. 2020 May 7;106(6):734–47. doi: 10.1016/j.ajhg.2020.04.006 (PMC7273533; doi:10.1016/j.ajhg.2020.04.006)
Supplement: Document S1. Figures S1–S5, Table S1, and Supplemental Methods [file mmc1.pdf]

The American Journal of Human Genetics, Volume *106*

Supplemental Data

Familial Hypocalciuric Hypercalcemia Type 1

and Autosomal-Dominant Hypocalcemia Type 1:

Prevalence in a Large Healthcare Population

Ridge Dershem, Caroline M. Gorvin, Raghu P.R. Metpally, Sarathbabu Krishnamurthy, Diane T. Smelser, Fadil M. Hannan, David J. Carey, Rajesh V. Thakker, Gerda E. Breitwieser, and on behalf of Regeneron Genetics Center

SUPPLEMENTARY APPENDIX

Supplemental Figures

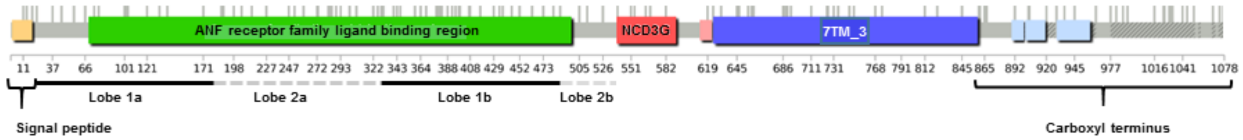

Figure S1. Domain locations of rare CaSR variants identified in the DiscovEHR cohort. The location of rare variants are indicated by upward gray ticks on the structure, and the major Pfam modules in the CaSR primary sequence are indicated by colored boxes. The yellow box indicates the location of the signal peptide, which is cleaved during CaSR biosynthesis. The extracellular domain (ECD) of CaSR contains an agonist binding region (green) and a cysteine-rich domain, NCD3G (red). The heptahelical transmembrane domain, 7TM_3 (blue) and carboxyl terminus (gray/light blue) span the membrane and facilitate signaling and localization, respectively. The lobes (1a, 2a, 1b, 2b) comprising the venus flytrap structure of the agonist-binding domain of the ECD are also indicated. Rare missense variants were evenly distributed through the domains, i.e., sixty-four were in the extracellular domain (ECD, residues 1-611; ~0.105 variants/residue), 22 were in the transmembrane heptahelical domain (TMD, residues 612-862; ~0.09 variants/residue), and 22 were in the carboxyl terminus (CT, residues 862-1078; ~0.102 variants/residue).

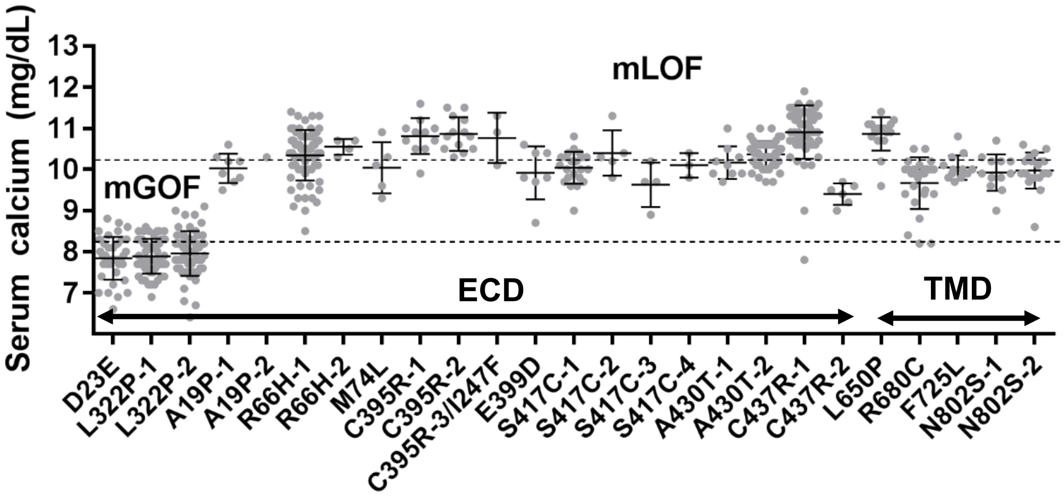

Figure S2. Total serum Ca^{2+} concentrations for all missense variants with potential ADH1 or FHH1 phenotypes. All total serum Ca^{2+} concentrations from the EHR were plotted for each individual having pLP or pP category variants. Mean ± S.D. that was within the outer ranges of normal (8.5-10.2 mg/dL were considered likely pathogenic variants and put into the functional analysis pipeline. ECD = extracellular domain; TMD = transmembrane heptahelical domain; mGOF = missense gain-of-function variants; mLOF = missense loss-of-function variants.

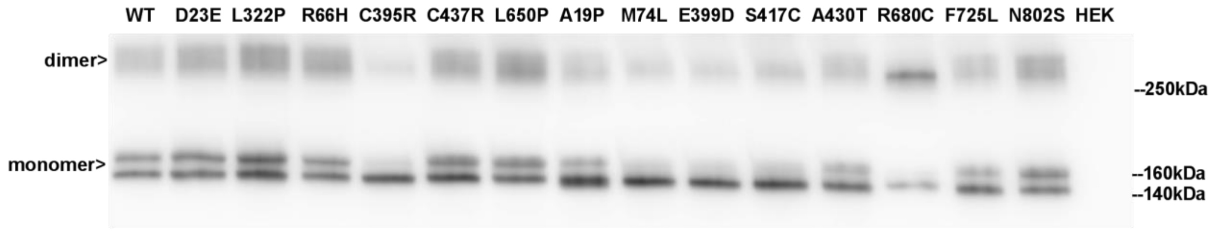

Figure S3. Representative western blots of CaSR mGOF and mLOF variants. WT CaSR or indicated variants were transiently expressed in HEK293 cells, cultured for 3 days, then lysed and 25 μg of lysates run on 4-15% polyacrylamide gels as detailed in Methods. Blots were probed with custom CaSR polyclonal antibody against the extracellular LRG epitope as previously described.[1]

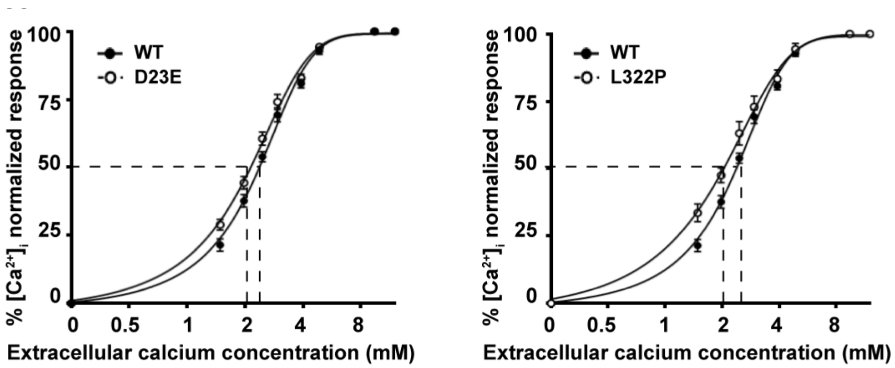

Figure S4. Activation of CaSR rare GOF variants by extracellular Ca^{2+}. Effects of D23E and L322P variants on Ca^{2+} signaling. Intracellular Ca^{2+} (Ca^{2+}_i) responses to changes in extracellular Ca^{2+} of HEK293 cells expressing WT or D23E or L322P variant CaSR proteins. The D23E and L322P variant CaSR proteins caused a leftward shift in the Ca^{2+}_e concentration-response curve with significantly reduced EC_{50} values compared to WT cells, consistent with a gain-of-function ($p<0.0001$, *F*-test). N=8-16 biological replicates. The Asp23Glu variant is predicted to alter signal peptide processing (SignalP4.1 Server).

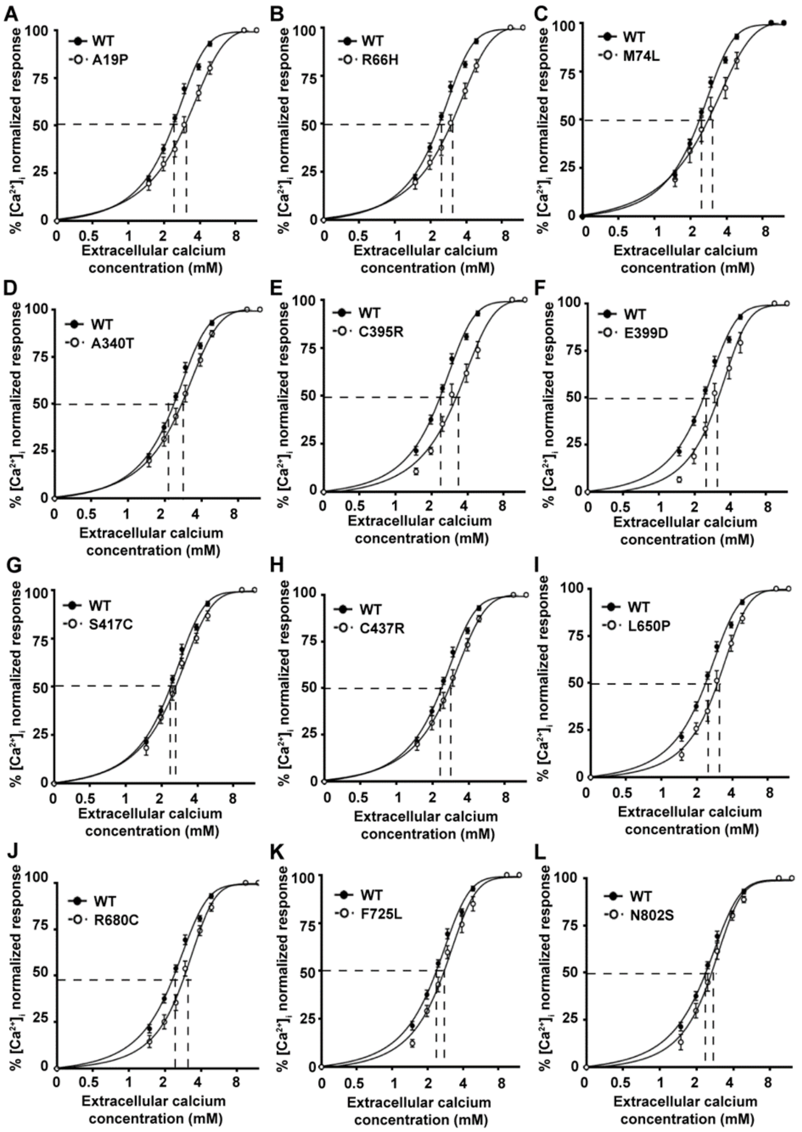

Figure S5. Activation of CaSR rare mLOF variants by extracellular Ca^{2+}. Ca^{2+}_i responses to changes in [Ca^{2+}_e] of HEK293 cells expressing WT or (**A**) A19P, (**B**) R66H, (**C**) M74L, (**D**)A340T, (**E**) C395R, (**F**) E399D, (**G**) S417C, (**H**) C347R, (**I**) L650P, (**J**) R680C, (**K**) F725L, (**L**) N802S variant CaSR proteins. The variant CaSR proteins caused a rightward shift in the Ca^{2+}_i concentration-response curve with significantly increased EC_{50} values compared to WT cells, consistent with a loss-of-function ($p<0.001$, *F*-test). N=8-16 biological replicates. The Ala19Pro variant is predicted to alter signaling peptide processing (SignalP4.1 Serve). Other mutations are

predicted to alter CaSR biosynthesis or trafficking (Cys394Arg, Glu399Asp, Cys437Arg). Phe725Leu is predicted to disrupt a highly conserved motif ^{798}PENFNEAK^{805} that contributes to G protein coupling in family C receptors.[2]

Supplemental Table

Basic Demographics	DiscovEHR Sequenced Patients[1]
N	51,289
Female, N (%)	30,290 (59)
Median Age, yr	61 (48-72)
Median BMI, kg/m^2	30(26-36)
Median Years of EHR data	14 (11-17)
Median medication orders/patient	129 (37-221)
Median lab results/patient	658 (197-1,119)
# PTs with median Ca^{2+}-albumin, N, (%)	42,466 (83%)

Table S1. Demographics and Clinical Characteristics of Individuals in the 51,289 DiscovEHR cohort. Abbreviations, EHR, electronic health record, BMI, body mass index.

[1]Values are expressed as median (interquartile range).

Supplemental Methods

A. In-silico Radical Mutations Pathogenicity (RMPath) prediction pipeline.

Rare variants are categorized into two groups: (1) nonsense and frame-shift variants, and (2) missense variants, and were scored by summing numerical values derived from predictions.

(1) Scoring of nonsense and frame-shift variants.

SnpEff fields: LOF[*].PERC ≥ 0.5 or NMD[*].NUMTR>3 are considered potential Loss of Function (LOF) variants.

(2) Scoring of missense variants.

1. SIFT scoring: D (damaging)=2; All others=0.

2. PolyPhen2 scoring: (i) HDIV: Probably damaging (D)=2; Possibly damaging (P)=1; all other results=0. (ii) HVAR: Probably damaging (D)=2; Possibly damaging (P)=1; all other results=0.

3. MutationTaster scoring: Disease causing (D)=2; all other results=0.

4. MutationAssessor scoring: High (H)=2; Medium (M)=1; all other results=0.

5. FATHMM scoring: D (damaging)=2; all other results=0.

6. LRT: D(deleterious)= 2; All others=0.

7. PROVEAN scoring: D(damaging)=2; all other results=0.

8. GRANTHAM scoring: ≥140=4; ≥120=3; ≥90=2; ≥70=1; all other results=0.

9. Genomic Evolutionary Rate Profiling GERP++ rejected substitutions (RS) scoring: ≥6=4; ≥5=3; ≥3.5=2; ≥2=1; all other results=0.

Summed scores from all tools generated the RMPath score, maximum possible value=24. Variants with RMPath scores ≤8 were predicted Benign (pB), scores from 6-11 were predicted Likely Benign (pLB), scores from 11-17 were predicted Likely Pathogenic (pLP) and those with scores ≥17 were predicted Pathogenic (pP). For clinical purposes, all variants are considered Variants of Unknown Significance (VUS) until family co-segregation and/or functional analyses *definitively established* a variant as pathogenic.

B. Intracellular Calcium Measurements.

Ca^{2+}_e-induced Ca^{2+}_i responses were measured by Fluo-4 Ca^{2+} assays recorded with a PHERAstar instrument (BMG Labtech) at 37°C, as previously described.[11] HEK293 cells transiently transfected with WT or variant CaSR were plated in poly-L-lysine treated black-walled 96-well plates (Corning). On the following day, cells were incubated in serum-free media for 2 hours, then loaded with Fluo-4 dye, prepared according to manufacturer's instructions (Invitrogen), for 60 minutes at 37°C. Baseline measurements were made and $CaCl_2$ was injected into each well to increase the extracellular [Ca^{2+}] in a stepwise manner from 0.5 to 10mM Ca^{2+}. Changes in intracellular Ca^{2+} were recorded on a PHERAstar instrument (BMG Labtech) at 37°C with an excitation filter of 485 nm and an emission filter of 520 nm. The peak mean fluorescence ratio of the transient response after each individual stimulus was measured using MARS data analysis software (BMG Labtech), and expressed as a normalized response. Nonlinear regression of concentration-response curves was performed with GraphPad Prism using the normalized response at each extracellular [Ca^{2+}] for each separate experiment to determine the EC_{50} (i.e., [Ca^{2+}_e] required for 50% of the maximal response). Assays were performed in 4 biological replicates for WT and variant CaSR. Statistical analysis was performed using the *F*-test.[3,4]

Web Resources

FATHMM, http://fathmm.biocompute.org.uk/

GERP++, http://mendel.stanford.edu/SidowLab/downloads/gerp/index.html

GRANTHAM, https://gist.github.com/danielecook/

LRT, http://www.genetics.wustl.edu/jflab/lrt_query.html

MutationAssessor, http://mutationassessor.org/r3/

MutationTaster, http://www.mutationtaster.org/

Pfam, http://pfam.xfam.org/search/#tabview=tab1

PolyPhen2, http://genetics.bwh.harvard.edu/pph2/

PROVEAN, http://provean.jcvi.org/index.php

SIFT, https://sift.bii.a-star.edu.sg/

SignalP 4.1 Server, http://www.cbs.dtu.dk/services/SignalP/

SnpEFF, http://snpeff.sourceforge.net/

References

1. Stepanchick, A., McKenna, J., McGovern, O., Huang, Y., Breitwieser, G.E. (2010) Calcium sensing receptor mutations implicated in pancreatitis and idiopathic epilepsy syndrome disrupt an arginine-rich retention motif. Cell. Physiol. Biochem. 26, 363-374.
2. Francesconi, A., Duvoisin, R.M. (1998) Role of the second and third intracellular loops in metabotropic glutamate receptors in mediating dual signal transduction activation. J. Biol. Chem. 273, 5615-5624.
3. Gorvin, C.M., Hannan, F.M., Cranston, T., Valta, H., Makitie, O., Schalin-Jantti, C., Thakker, R.V. (2017) Cinacalcet rectifies hypercalcemia in a patient with familial hypocalciuric hypercalcemia type 2 (FHH2) caused by a germline loss-of-function Galpha11 mutation. J. Bone Miner. Res. 33, 32-41.
4. Nesbit, M.A., Hannan, F.M., Howles, S.A., Babinsky, V.N., Head, R.A., Cranston, T., Rust, N., Hobbs, M.R., Heath, H. 3rd, Thakker, R.V. (2013) Mutations affecting G-protein subunit α11 in hypercalcemia and hypocalcemia. N. Engl. J. Med. 368, 2476-2486.
